# Rigid cystoscopy *versus* flexible outpatient cystoscopy: the economic cost behind the epidemiological and social impact

**DOI:** 10.31744/einstein_journal/2026GS1136

**Published:** 2026-06-08

**Authors:** Guilherme Andrade Peixoto, Matheus Pascotto de Salles, Edson Amaro, Fernando Korkes

**Affiliations:** 1 Centro Universitário FMABC Santo André SP Brazil Centro Universitário FMABC, Santo André, SP, Brazil.; 2 Hospital Israelita Albert Einstein São Paulo SP Brazil Hospital Israelita Albert Einstein, São Paulo, SP, Brazil.

**Keywords:** Cystoscopy, Urinary bladder neoplasms, Costs and cost analysis, Health policy, National health programs

## Abstract

**Introduction:**

The Brazilian Public Health System predominantly performs cystoscopy in hospitals using rigid cystoscopes in operating room settings. This approach results in high operational costs and scheduling delays associated with hospital admission and preoperative protocols. Flexible cystoscopy can be performed in an outpatient setting and may substantially reduce both costs and waiting times.

**Objective:**

To compare the costs and waiting times associated with rigid and flexible cystoscopy in São Paulo, Brazil.

**Methods:**

A retrospective review at a municipal hospital in Brazil identified patients who underwent diagnostic rigid cystoscopy. The analysis estimated the cost of the procedure and the time from indication to examination. Because no public hospital performed flexible cystoscopy during the study period, fees from private hospitals were collected as a proxy and incorporated into a one-way sensitivity analysis.

**Results:**

The mean cost per rigid cystoscopy reached BRL 8,319.45 (US$1,618.82), ranging from BRL 6,187.10 (US$1,203.90) to BRL 10,512.34 (US$2,045.52). Costs were higher when biopsy occurred (mean BRL 9,744.20) and lower when biopsy was not performed (mean BRL 6,609.75). Fees for flexible cystoscopy ranged from BRL 1,523.81 (US$296.51) to BRL 2,300.00 (US$447.54), with a mean value of BRL 1,800.60 (US$350.37). The mean interval from indication to procedure was 55.64 days for rigid cystoscopy compared with 6.34 days in centers that perform flexible cystoscopy. In sensitivity analysis, even when public-sector flexible cystoscopy costs reached 1.5-3.0× the private fee (BRL 2,701-5,402), the procedure remained approximately 35-68% less expensive than rigid cystoscopy.

**Conclusion:**

Management of bladder cancer imposes a substantial economic burden on Brazilian Public Health System. Despite the asymmetry between public and private data sources, these findings support adoption of outpatient flexible cystoscopy to accelerate diagnosis and reduce costs, with important implications for health policy.

## Highlights

■ Rigid cystoscopy requires greater resources and generates higher overall costs.

■ Flexible cystoscopy substantially shortens the waiting time for diagnosis.

■ Cost advantages of flexible cystoscopy persist even under conservative assumptions.

## In Brief

Peixoto et al. conducted a retrospective cost analysis at a Brazilian public oncology hospital comparing operating room-based rigid cystoscopy with outpatient flexible cystoscopy. Rigid cystoscopy cost BRL 8,319 with a mean waiting time of 56 days, whereas flexible cystoscopy averaged BRL 1,801 with a mean waiting time of six days. Sensitivity analyses demonstrated persistent cost savings with flexible cystoscopy.

## Graphical abstract

**Figure:**
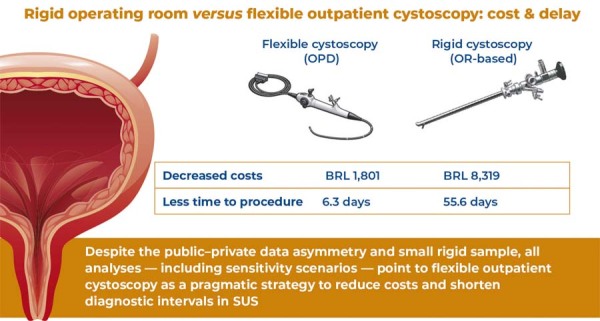

## INTRODUCTION

Bladder cancer ranks as the eleventh most commonly diagnosed cancer worldwide when considering both sexes.^(1,2)^ It is also among the most costly cancers for both public and private health sectors globally.^(3-5)^ In the United States, bladder cancer has been estimated to be the most expensive cancer, with annual costs of approximately US$9 billion.^(6,7)^ This amount is six times higher than the total expenditure on all cancer treatments in Brazil combined.^(8)^ Given the substantial risk of recurrence (30-70%) and progression (10-30%) following tumor resection, patients require frequent cystoscopic and cytological surveillance, potentially throughout life.^(9,10)^

Survival in bladder cancer depends strongly on stage at diagnosis and the interval to diagnosis and treatment.^(6,11)^ Advanced disease often requires longer hospital stays and additional diagnostic tests and is associated with more complications compared with other cancers.^(12)^ Within the Brazilian Public Health System (SUS - *Sistema Único de Saúde*), referral to urology typically requires a “strong suspicion” of bladder cancer, which can create delays related to administrative bottlenecks, waiting lists, and the need for complementary examinations. Because definitive diagnosis relies on cystoscopy performed by a urologist, primary and secondary care settings cannot confirm the disease through biopsy, which may further prolong the diagnostic process.^(13,14)^

The location and technique of cystoscopy depend primarily on the available equipment. Rigid cystoscopy generally requires an operating room (OR) and anesthesia and involves costly infrastructure. When tumors are identified, same-session resection may be possible; however, when no tumor is present, use of the OR may represent inefficient resource allocation. In contrast, flexible cystoscopy can be performed in the outpatient setting using topical intraurethral local anesthesia. Although the initial purchase of flexible equipment and establishment of an outpatient setup may involve higher upfront costs, long-term per-procedure costs may be lower.

Within SUS, cystoscopy is predominantly performed using rigid equipment in the OR, which entails high operational costs and delays related to pre-anesthetic scheduling. Because bladder cancer progression is time-sensitive,^(11,15)^ reducing the diagnostic interval is both clinically and economically important. Flexible cystoscopy may reduce costs and waiting times while increasing the likelihood of early diagnosis.

## OBJECTIVE

This study aimed to quantify and compare the direct costs and scheduling intervals of diagnostic cystoscopy performed as rigid, OR-based procedures in a public hospital *versus* flexible outpatient procedures in São Paulo, Brazil. The primary endpoints were the mean per-procedure cost (BRL) and the mean time from urologist indication to procedure (days). Secondary objectives included assessing cost variation associated with concomitant biopsy, describing the major cost components, and conducting a one-way sensitivity analysis that varied hypothetical public-sector flexible cystoscopy costs (1.0-4.0× the mean private fee) against observed rigid cystoscopy costs. The study aimed to provide locally grounded evidence to inform policies within Brazilian Public Health System regarding the potential shift of diagnostic cystoscopy from OR-based procedures to outpatient pathways.

## METHODS

A cost analysis of all patients treated for bladder cancer from November 2019 to November 2020 was conducted at *Hospital Municipal Gilson de Cássia Marques de Carvalho*, a public oncology hospital managed by *Hospital Israelita Albert Einstein* through a public-private partnership. The study received approval from the local ethics committee (CAAE: 40836920.0.0000.0071; #4.986.464).

Patients who underwent diagnostic cystoscopy alone were included. Cystoscopies performed in association with other procedures, such as transurethral resection of bladder tumor, were excluded because isolating cystoscopy-specific costs in these cases was difficult. All costs related to rigid cystoscopy were calculated**,** including admission, preoperative examinations and evaluations, anesthesia, professional fees, and management of complications.

Because no public municipal hospital in São Paulo performed flexible cystoscopy during the study period, private hospitals were surveyed to obtain their fees. These values represent market prices rather than the actual cost of performing the procedure.

A cost-minimization analysis was conducted because the literature indicates comparable effectiveness and safety for flexible and rigid cystoscopy.^(16-18)^ To address the asymmetry between public and private data sources, a one-way sensitivity analysis varied a hypothetical public-sector cost for flexible cystoscopy from 1.0× to 4.0× the mean private fee (BRL 1,800.60). Percent savings were then calculated relative to the observed mean cost of rigid cystoscopy (BRL 8,319.45).

Statistical analysis used STATA 14.0 (StataCorp LP, College Station, TX, USA). Costs were reported in BRL, with conversion to US dollars based on the average exchange rate between November 2019 and November 2020 (US$1=R$5.1392).

## RESULTS

During the study period, 107 patients received treatment for bladder cancer; 11 (10.2%) underwent diagnostic rigid cystoscopy alone without additional treatment. Demographic characteristics appear in table 1.

**Table 1:** Demographic characteristics of patients who underwent rigid cystoscopy

	Rigid cystoscopy
n	11
Age (mean, years)	67.5
Sex - n (%)	
Male	8 (73)
Female	3 (27)
Comorbidities (%)	
Vascular or heart disease	9
Hypertension	55
Diabetes mellitus	18
Second malignancy	18
Smoking (current or former)	55
Other	9

The mean cost per rigid cystoscopy was BRL 8,319.45 (US$1,618.82), with a range of BRL 6,187.10-10,512.34. Six procedures (55%) included biopsy, with a mean cost of BRL 9,744.20, whereas five procedures (45%) did not include biopsy, with a mean cost of BRL 6,609.75.

Flexible cystoscopy fees in private hospitals in São Paulo ranged from BRL 1,523.81 to BRL 2,300.00, with a mean value of BRL 1,800.60. These procedures occurred in the outpatient setting under local anesthesia and did not require hospital admission.

The mean time to rigid cystoscopy reached 55.64 days (range 9-103) from the time of indication to the procedure. In centers that offer flexible cystoscopy, the mean scheduling interval reached 6.34 days (range 5-7). Table 2 summarizes these data.

**Table 2:** Data related to cost and time to cystoscopy execution

Cystoscopy	n (%)	Mean cost (BRL)	Variation (min-max) (BRL)	Follow-up (months)	Mean time to execution (days)*
Rigid	11 (100)	8,319.45	6,187.10-10,512.34	10.7	55.7
With biopsy	6 (55)	9,744.20	8,455.22-10,512.34	-	55.2
Without biopsy	5 (45)	6,609.75	6,187.10-8,772.60	-	56.2
Flexible	-	1,800.00	1,523.81-2,300.00	-	6.0

* From cystoscopy indication to execution.

A one-way sensitivity analysis assumed public-sector flexible cystoscopy costs equal to 1.0×, 1.5×, 2.0×, 3.0×, and 4.0× the mean private fee (BRL 1,800.60). Estimated savings relative to the observed mean rigid cystoscopy cost (BRL 8,319.45) reached approximately 78.4%, 67.5%, 56.7%, 35.1%, and 13.4%, respectively (Table 1S, Supplementary Material).

## DISCUSSION

Each year, nearly ten thousand new cases of bladder cancer are diagnosed in Brazil,^(19)^ with a substantially higher prevalence because the disease often follows a chronic course that requires lifelong surveillance. A previous study estimated that 43% of bladder cancer-related costs arise from diagnosis and surveillance,^(10)^ highlighting the central role of cystoscopy.

At our public hospital, 11 OR-based diagnostic rigid cystoscopies performed without subsequent treatment generated a total cost of BRL 91,513.92. If these procedures had occurred through flexible outpatient cystoscopy, costs and waiting times would likely have been considerably lower. Using private-sector fees as a proxy, the mean cost of flexible cystoscopy reached BRL 1,800.60 compared with BRL 8,319.45 for rigid cystoscopy. Sensitivity analysis indicates that the cost advantage would persist even if public-sector flexible cystoscopy costs were substantially higher than private-sector fees.

Rigid cystoscopy required a mean interval of 56 days from indication to execution, largely reflecting OR queues and pre-anesthetic scheduling pathways, whereas centers that offer flexible cystoscopy scheduled procedures within approximately six days. Given the time-sensitive nature of bladder cancer,^(20)^ shortening the diagnostic interval carries important clinical implications.

A key limitation involves methodological asymmetry. Rigid cystoscopy values reflect actual public-sector costs, whereas flexible cystoscopy figures represent private-sector fees. This discrepancy limits direct comparability. Private prices may include profit margins, while public implementation would also require investment in start-up, maintenance, and reprocessing infrastructure that market fees do not fully capture.

The rigid cystoscopy cohort remains small (n=11) and includes only diagnostic procedures without transurethral resection of bladder tumor. This sample may not represent the full clinical spectrum and increases the risk of selection bias. The single-center design also limits generalizability.

The short follow-up period, which reflects early service maturation, prevents robust long-term cost projections. Future evaluations with longer follow-up periods will become necessary as the outpatient flexible cystoscopy pathway becomes more established.

Although indirect costs fall outside the scope of the present cost analysis, factors such as productivity losses associated with hospitalization and longer waiting times, caregiver time, travel expenses, and quality-of-life effects likely further strengthen the economic advantages of outpatient flexible cystoscopy.

From a health policy perspective within SUS, priority actions include procurement of flexible cystoscopes with service and maintenance contracts, training and workflow standardization for outpatient cystoscopy and instrument reprocessing, alignment of reimbursement policies to recognize outpatient cystoscopy and encourage a shift from OR-based diagnostic procedures, and establishment of referral pathways for hematuria with defined time targets. Key barriers include upfront capital investment, infection control logistics, and change management. Pilot implementation at reference centers may reduce risk before broader scale-up.

Recent reductions in device prices and improvements in high-definition imaging have addressed earlier concerns regarding flexible cystoscopes. Current evidence supports clinical equivalence between flexible and rigid cystoscopy for diagnostic purposes.^(16-18)^

## CONCLUSION

Despite the asymmetry between public and private data and the small rigid cystoscopy sample, all analyses, including the sensitivity scenarios, indicate that outpatient flexible cystoscopy represents a pragmatic strategy to reduce costs and shorten diagnostic intervals within Brazilian Public Health System. Given the substantial economic burden associated with bladder cancer, policy measures that enable outpatient flexible cystoscopy and streamline the evaluation of hematuria may provide immediate value.

## SUPPLEMENTARY MATERIAL

SUPPLEMENTARY MATERIAL
