# Supplementary material for: Rigid cystoscopy versus flexible outpatient cystoscopy: the economic cost behind the epidemiological and social impact
Source: Einstein (Sao Paulo). 2026 Jun 8;24:eGS1136. doi: 10.31744/einstein_journal/2026GS1136 (PMC13399425; doi:10.31744/einstein_journal/2026GS1136)
Supplement: SUPPLEMENTARY MATERIAL [file 2317-6385-eins-24-eGS1136-suppl01.pdf]

SUPPLEMENTARY MATERIAL

Rigid cystoscopy *versus* flexible outpatient cystoscopy: the economic cost behind the epidemiological and social impact

Guilherme Andrade Peixoto, Matheus Pascotto de Salles, Edson Amaro Junior, Fernando Korkes

DOI: 10.31744/einstein_journal/2026GS1136

Table 1S. One-way sensitivity analysis of hypothetical public-sector flexible cystoscopy costs compared with the observed rigid cystoscopy cost (BRL 8,319.45)

Assumed public flexible cost	BRL	Estimated savings vs. rigid (BRL)	% savings
1.0× private fee	1,800.60	6,518.85	78.4
1.5× private fee	2,700.90	5,618.55	67.5
2.0× private fee	3,601.20	4,718.25	56.7
3.0× private fee	5,401.80	2,917.65	35.1
4.0× private fee	7,202.40	1,117.05	13.4
